# THE MEDIATING ROLE OF ADVANCE CARE PLANNING IN ACCULTURATION AND USE OF NURSING HOME AMONG KOREAN AMERICANS

**DOI:** 10.1093/geroni/igz038.1909

**Published:** 2019-11-08

**Authors:** Soyeon Cho

**Affiliations:** City University of New York-New York City College of Technology, Brooklyn, New York, United States

## Abstract

The study examined attitudes towards Advance Care Planning (ACP) as a potential mediator in the association between acculturation and willingness to use nursing home in Korean American older adults (aged 60 and older). Data were driven from a cross-sectional study of 235 community-dwelling Korean American older adults (aged 60 and older) in 2013. Multivariate regression models of willingness to use nursing home were entered in the following order: (1) demographics, (2) health, (3) acculturation, and (4) attitudes towards advance care planning. The mediation effect of attitudes towards ACP in the relationship between acculturation and willingness to use nursing home was separately examined using the bootstrapping method. Higher acculturation was associated with positive attitudes towards ACP and more likelihood of using nursing home. The proposed mediation model was fully supported: positive attitudes towards ACP served as an intervening step between acculturation and willingness to use nursing home. The mediating role of attitudes towards ACP yields implications for developing culturally sensitive advance care planning education program targeting older individuals.

